# Supervised deep learning for real-time quality monitoring of laser welding with X-ray radiographic guidance

**DOI:** 10.1038/s41598-020-60294-x

**Published:** 2020-02-25

**Authors:** Sergey Shevchik, Tri Le-Quang, Bastian Meylan, Farzad Vakili Farahani, Margie P. Olbinado, Alexander Rack, Giulio Masinelli, Christian Leinenbach, Kilian Wasmer

**Affiliations:** 1 Laboratory for Advanced Materials Processing (LAMP), Swiss Federal Laboratories for Materials Science and Technology (Empa), Thun, Switzerland; 2 Coherent Switzerland, Belp, CH-3125 Switzerland; 3 ESRF – The European Synchrotron, Grenoble, France

**Keywords:** Laser material processing, Computational science

## Abstract

Laser welding is a key technology for many industrial applications. However, its online quality monitoring is an open issue due to the highly complex nature of the process. This work aims at enriching existing approaches in this field. We propose a method for real-time detection of process instabilities that can lead to defects. Hard X-ray radiography is used for the ground truth observations of the sub-surface events that are critical for the quality. A deep artificial neural network is applied to reveal the unique signatures of those events in wavelet spectrograms from the laser back-reflection and acoustic emission signals. The autonomous classification of the revealed signatures is tested on real-life data, while the real-time performance is reached by means of parallel computing. The confidence of the quality classification ranges between 71% and 99%, with a temporal resolution down to 2 ms and a computation time per classification task as low as 2 ms. This approach is a new paradigm in the digitization of industrial processes and can be exploited to provide feedbacks in a closed-loop quality control system.

## Introduction

The introduction of laser technology in metal welding of metals is dated back to the late 1960s^1,2^ when it immediately showed advantages as compared to traditional arc welding^3^. The attractions of this technique are in the non-contact processing, the absence of tool wear, high aspect ratio of the melt pool, better material fusion, possibility to process refractory materials, low running costs and high processing speed^3,4^. Today, laser welding is a key technology in many fields e.g. automotive^5^ and aerospace^3,6^ industries, naval and heavy machinery production^7^, medicine and micromechanics^3^. Unfortunately, the potential of this technology is not fully exploited, particularly in applications that require the guarantee of high weld quality. The reason is the non-linear nature of light-matter interactions, which complicates the reproducibility of the weld quality in mass production^8–10^. The complex dynamics of the process, especially in keyhole welding regime, and its instabilities can cause various defects at the joint^3,10–12^. A defect type of particular interest is porosity, which is a hidden threat for the mechanical properties of the workpieces^3,9–11^. Obviously, an adequate, robust and low cost quality monitoring system is of great desire. The major challenge in developing such technique is in the difficulties to inspect directly the sub-surface behavior of the process zone in real-life conditions^13^.

Multiple approaches have been proposed, which are mostly based on mathematical modeling aiming to reconstruct the under surface dynamics using inspections of the surface via measurements of temperature^11,12,14,15^, optical^16,17^ and/or acoustic^18,19^ emissions (AE). However, those approaches face three main problems. Firstly, modeling often suffers inaccuracies originating from the deviations of the model assumptions from the real parameters’ values. More complicated assumptions can be used to improve the accuracy, but, at the same time, they significantly increase the computation requirements. Secondly, modeling is rather time-consuming and thus is not suitable for real-time monitoring. Finally, the proposed sensors are mostly sensitive to the surface region of the melt pool, while critical events like pore formation take place deep inside the process zone^11,20^. Therefore, the link between the obtained signals and the weld quality is not straightforward^13,21^.

Recently, another approach for *in situ* and real-time monitoring of laser welding was reported by the present authors^22^. Its novelty was in the combination of advanced signal processing and machine learning (ML) techniques to analyze the signals. In particular, wavelet decomposition and Laplacian support vector machine were applied to the AE and the optical signals acquired during the welding process. These sensors were chosen because they required low cost hardware and they have been implemented in various industrial applications^22^. The exploited ML techniques allowed retrieving unique features for different weld qualities, which were later used for *in situ* monitoring. In addition, the computation time was as low as 70 ms, making that approach suitable for real-time operation.

The present work aims at exploiting the previously reported approach for a more challenging task, namely, to classify the momentary events during laser welding process, which have a significant influence on the weld quality. In order to achieve this objective, it is critical that the relationships between the events and the sensor signals are established properly. Therefore, high-speed hard X-ray radiography was used to observe *in situ* the process zone with very high spatial and temporary resolutions^23,24^. The critical events were then determined from the X-ray videos and the signals, which correspond to those events, were extracted accordingly. State-of-the-art ML algorithm, namely, deep convolutional neural network (CNN) was employed to investigate the existence of the unique signatures in the laser back reflected (LBR) and AE signals that were recorded during the welding process. CNN is known for its high efficiency in structuring statistical data and these capabilities were fully exploited in this work^25,26^. To improve the real-time performance of the system, graphics processing units (GPU) with parallel computing were used to decrease the computation time^27^, making this solution of high readiness for further practical usage.

## Results and Discussion

### X-ray radiography of laser welding

**Figure 1:**
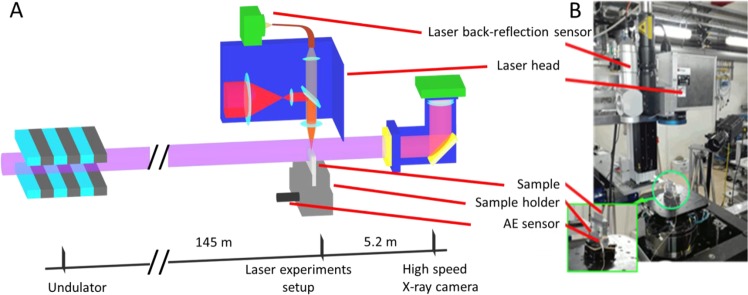
(**A**) Schematic of the experimental setup for *in-situ* X-ray radiography of the laser welds, adapted from^29^. The bar below defines the key nodes of the setup and their mutual positions; (**B**) Picture of the welding experimental station.

High-speed X-ray radiography was used to visualize the dynamical behavior of the melt pool directly inside the workpiece during the laser welding process. These observations were essential to establish the ground truth of the events that were employed to define the different categories of quality. The experiments were carried out at the imaging beamline ID19 at the European Synchrotron (ESRF) which provides ultra-high temporal X-ray imaging resolution^23,24^. In particular, X-ray phase contrast imaging was employed to enhance the sensitivity, especially at the boundaries between phases (solid, gas and vapor), where the X-ray beam refraction is the highest^28^. To collect radiographic images of the welding process, a specific setup was developed and its general schematic is presented in Fig. 1A. The distance from the X-ray source to the sample was 145 m, and 5.2 m from the sample to the detector. The X-ray beam was provided by a U-13 type undulator with a minimum gap size of 11.1 mm. The beam was attenuated by an aluminum filter and a diamond attenuator of 1.4 mm thickness, resulting in a *pink* beam with averaged power of about 26 keV. Additionally, the beam exposure was regulated by a shutter in order to illuminate the sample only during recordings. To avoid saturating the camera, the electronic shutter of the camera was set at an exposure time of 30 µs. Two slit systems allowed adjusting the beam’s spatial field of view to the one needed to cover the laser welding process zone and to match with the image size on the high-speed camera sensor. The radiographic imaging of the laser welding process was recorded by an indirect X-ray detector, composed of a 250 μm thick Ce-doped LuAG (Lu_3_Al_5_O_12_) scintillator. A mirror directed the scintillator’s re-emittance to the high-speed camera, which was coupled with an objective and placed at 90° angle relatively to the X-ray beam as shown in Fig. 1A. The experimental recording image rate was fixed at 28,762 fps, while the effective pixel size of the X-ray image detector was 11 μm.

A picture of the laser welding station is presented in Fig. 1B. The station included a single mode fiber laser source StarFiber 150 P (Coherent Inc., Switzerland), with a wavelength of 1070 nm and a maximum peak power of 1.5 kW. The light from the laser source was transmitted to a laser head via an optical fiber with a core diameter of 12 µm. The laser head focused the light on the sample surface into a spot of 30 µm diameter at 1/*e*^2^ of the beam’s maximum intensity using an *f*-theta lens with a 170 mm focal length. The laser head was additionally equipped with an optical system for collecting the radiations emitted/reflected from the process zone. The built-in germanium (Ge) photodiode originally had a spectral sensitivity in the range of 800–1,800 nm. A narrow band-pass filter (FB1070-10, Thorlabs Inc., USA) with a center wavelength of 1070 ± 2 nm and a Full Width Half Max (FWHM) of 10 ± 2 nm was installed to the photodiode to provide a selective transmission of the LBR radiation from the process zone^22,29–31^. The photodiode signal was acquired with an oscilloscope Teledyne LeCroy HDO6104 at sampling rates within the range of 50–2,000 kHz. Depending on the duration of the experiments, the sampling rate was adjusted accordingly so that the whole signal did not exceed the internal memory of the oscilloscope. The acquisition of the LBR signal was triggered by the electrical output from the laser source, which was switched to 5 V immediately after the start of laser emission with a maximum delay of 20 µs.

Laser welding experiments were performed on rectangular plates of AA5005 aluminum alloy (98.1% Al, 0.9% Mg and 1% of other elements) with a thickness of 2 mm. This material was chosen for its relatively low *Z*-number, which allowed the transmission of 60% of the incident X-ray beam, directed perpendicular to the sample surface as indicated in Fig. 1A. The selected thickness with the simultaneous adjustment of the beam intensity provided a sufficient contrast in X-ray images. The samples were fixed in a sample holder placed on an *XYZ* table (Fig. 1A,B). The material of the sample holder was identical to the samples and both had tight contact during the experiment. This setup provided minimal losses of AE signals at the interfaces. The AE sensor was attached to the sample holder in the same position throughout all the experiments. The AE sensing was carried out with a piezo sensor PICO HF-1.2 (Physical Acoustics, Germany). The signals were recorded with a data acquisition unit from Vallen (Vallen Gmbh, Germany) at a fixed sampling rate of 10 MHz. The AE acquisition was triggered as the AE signal itself reached a threshold level, which was defined by preliminary tests.

During welding experiments, the *XYZ* table was either kept stationary or translated at a constant velocity of 1.5 mm/s in the direction perpendicular to both the laser and X-ray beams, allowing spot and seam welds, respectively. Simultaneously, the relative positions of the laser and X-ray beams were kept stationary and were centered in the field of view of the X-ray camera. The welding experiments were carried out in pulsed mode with a repetition rate of 10 Hz with pulse durations and output laser powers within the ranges 10–15 ms and 1–1.5 kW, respectively. The combinations of these process parameters were chosen to provoke different welding qualities.

### Signal processing

The analysis of the acquired signals aimed at searching for unique signatures of different pre-defined physical events. It involved two techniques, namely: i) wavelet packet transforms (WPT) and ii) deep learning. WPT was used to substitute the collected LBR and AE signals by wavelet spectrograms, formed as relative energies of the narrow frequency bands^32,33^. More details of this technique can be found in our previous works^22,30,34,35^. Such signal representation has four major advantages. Firstly, it reduces significantly the computational data. Secondly, the de-noising of the signals is performed by selecting the specified frequency bands^36^. Then, it allows the interpretation of signals as an evolution of the chosen frequency bands in time. Finally, it adapts the signals so that they can be used as input of already existing CNN realizations. Indeed, most of the existing CNNs are developed for image processing, where the 2D spatial domain of an image is analyzed. The domain of wavelet spectrograms is also a 2D time-frequency domain, to which existing CNN can be applied directly. At the same time, in practical applications, the spectrograms preserve the special features of the original signals, and evidence of this was successfully demonstrated in earlier investigations^22,30,31,34,35^.

The choice of wavelet base for the spectrogram computations is a critical task. In this study, an exhaustive search was carried out over different known wavelet families (Symlets, Daubechies, Coiflets)^36^ to find the optimum. The choice for the optimal criterion was the minimum wavelet approximation error of the given original LBR and AE signals. Based on the search, Daubechies wavelet with ten vanishing moments was found to fit best the collected data^22^.

**Figure 2:**
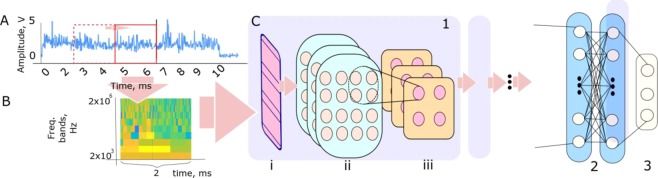
(**A**) An example of LBR signal acquired during the process; (**B**) construction of the wavelet spectrograms from the LBR signal; (**C**) scheme of the CNN used for the analysis of the data including: (1) self-feature extraction block, (2) fully connected layers and (3) softmax layer. The self-feature extraction block has three elements, namely (i) convolution layer, (ii) features map layers and iii) pooling layers.

In this study, the LBR and AE signals were referenced to X-ray radiographic data and patterns were firstly extracted that corresponded to different quality-significant events. The synchronization of the signals with the X-ray video was made with respect to the sampling rates of the signal acquisition units and the X-ray camera. Furthermore, it must be emphasized that the timestamp *t* = 0 ms in both signals coincided with the start of the laser emission. The latter could also be observed in the X-ray videos as the start of melting/modification at the sample’s surface. The extracted patterns were then scanned by a running window with a fixed time span. The computation of the spectrograms for each window (pattern) was carried out individually and was used as input data for the CNN. The time span of the running window defined the temporal and spatial resolutions of the quality estimates knowing the laser scanning velocity. The shifts of the running window in the time domain allowed real-time tracking of the process and are schematically displayed in Fig. 2A. The window is represented by a red rectangular with dash and solid lines indicating two consequent patterns, bounded by the running window. The spectrograms of each individual pattern from the LBR and AE were built as shown in Fig. 2B, and grouped according to the corresponding quality-significant events. Based on the results of our previous works^22,30^, 4096 frequency bands from each spectrogram were extracted and fed to the CNN. The wavelet transformation was tuned so that the spectrograms had the same size, regardless of the number of data points in the patterns. This step was necessary for the spectrograms to be processed by the CNN.

The deep learning framework was applied to the LBR and AE wavelet spectrograms to search for the presence of stable signatures that are unique for the predefined quality-significant events. The mathematical formulation of such a search is well developed in statistical learning, where the differences over statistical datasets are disclosed and a specialized function is constructed to enhance the separation of the data through a series of non-linear transformations. In this work, we exploited fully the recent advances in this field. In particular, state-of-the-art deep convolutional neural networks (CNN) were selected due to their high efficiency in data structuring^23,24^.

CNN is an extension of traditional neural networks and a general architecture can be seen in Fig. 2C. The CNN operational principles were inspired by the processing of visual information in the mammalians cortex^37^, where different neuronal assembles respond to only particular stimuli, localized in the visual field. In CNN, this is formalized by introducing a convolution operation (Fig. 2C, 1, i), where the spatial domain of the input data is scanned by a set of spatially localized filters. Similar to the visual cortex, each local filter of the CNN is sensitive to a specific pattern, which is encoded into the artificial neuronal structures. The full ensemble of local filters, applied to the entire spatial domain of the input, provides the global data filtering. This filtering approach selects the unique combinations of specific patterns, while the entire convolution operation is performed inside the convolution layer (Fig. 2C, 1, i). The responses of all local filters after convolution are stored inside the feature maps as presented in Fig. 2C, 1, ii. The on-growing amount of the information after each convolution can be reduced by introducing a pooling layer, which decreases the resolution of the feature maps by merging them as shown in Fig. 2C, 1, iii^26^. We applied this technique to each feature map.

**Figure 3:**
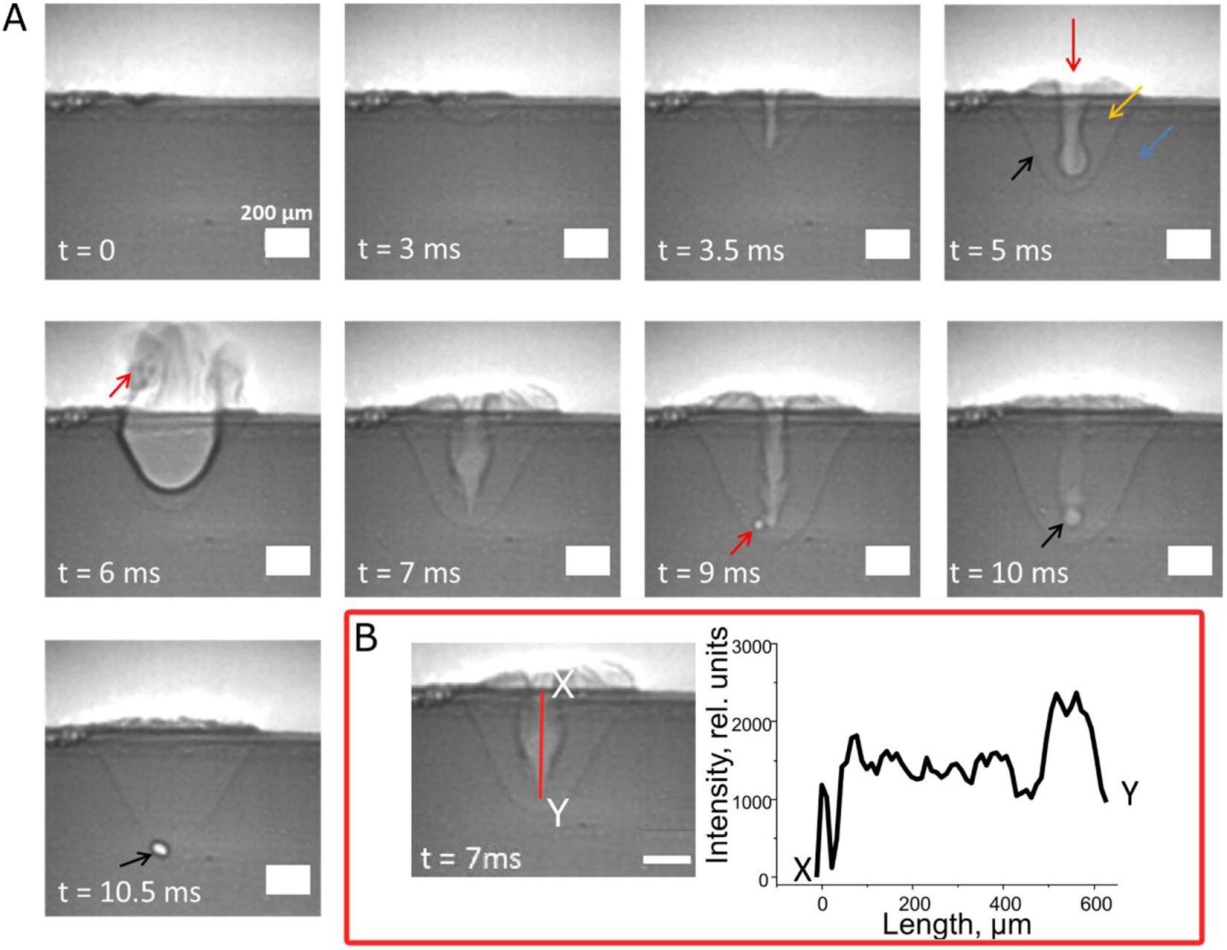
(**A**) Typical X-ray snapshots of the aluminum sample exposed to a single laser pulse of 10 ms pulse duration at a power of 1 kW. The sample was shifted relatively to the laser beam with a constant velocity of 1.5 mm/s. The time stamps are counted from the start of the laser irradiation. The scale bar at bottom right defines a distance of 200 µm. The red, orange, blue and black arrows at *t* = 5 ms mark the gas phase, liquid phase, solid phase and the liquid/solid boundary, respectively. The red arrows at *t* = 6 ms and *t* = 9 ms show the molten material expelled during blowout and the pore formed during unstable keyhole, respectively. The black arrows at *t* = 10–10.5 ms show the pore formed by the rapid collapse of the keyhole at the end of the irradiation; (**B**) Pixel intensity profile of the keyhole channel. The brighter pixels correspond to regions with lower density^28^.

A group from each convolution layer, feature map, and pooling layer composes a self-feature extraction block (Fig. 2C, 1) of the CNN. Compared to the regular neural networks, this provides a better capability to search for the most representative patterns in the data^28,29^. It is carried out by tuning the inner parameters (i.e. neuronal weights) of the local filters in the convolution layers during training. The sequence of the self-feature extraction blocks (Fig. 2C, 1) provides a multiscale data analysis. The output of the self-feature extraction blocks can then be classified in a regular, fully connected network (Fig. 2C, 2). In the present study, one hidden layer was used for this purpose, while the output was observed after a final softmax layer (Fig. 2C, 3)^29^.

In this work, three models were tested, covering both conventional and state-of-the-art CNN architectures, namely: (i) temporal^25^, (ii) conventional^26^ and (iii) cross-residual CNNs^38^. In particular, temporal CNNs have recently attracted a lot of attention due to the possibility to capture the temporal dependencies in sequential data. Specifically, temporal CNNs take a sequence of any length as input and map it to the sequence of the same length at output. The model uses causal convolutions, in which the output at a specific time stamp is also dependent on the elements from the preceding time stamp, stored in another layer. On the contrary, cross-residual CNNs enables intuitive learning across multiple related tasks using cross-connections called cross-residuals. The structure in Fig. 2C was fed with the wavelet spectrograms and the local filters convolved over their time-frequency domain. The output was a unique label of the corresponding quality-significant event, represented by the input spectrogram. In this context, the interpretation of the unique signatures can be easily understood as the combinations of different wavelet bands with a specific time evolution.

The processing parameters of the procedure shown in Fig. 2 include three main variables. These are: (1) the time span of the running window of the wavelet spectrograms, (2) the number of the self-feature extracted layers (Fig. 2C, 1) and 3) the convolutions’ spatial size (Fig. 2C, 1, i). The time span of the running window was estimated based on the duration of the transient of the LBR and AE signals. It is known that laser welding is accompanied by phenomena with durations between 0.2 ms and 10 ms^3,11,12,19,39^. Consequently, several time spans were tested and the highest accuracy was reached with the time span of 2 ms.

The parameters of the CNN model were not obvious at the beginning of the work and the tuning was performed to reach the highest accuracy rates. The exact model’s structures for each CNN are given in the materials and methods section. The computation time for a single classification task was 2 ms using an un-optimized code.

### Definition of the quality-significant events using X-ray radiography

Figure 3A shows an example set of X-ray snapshots obtained from a process with a laser pulse duration of 10 ms and a laser power of 1 kW. This experimental condition was sufficient to provoke different major quality-significant events, which are described in detail in the following paragraphs. Different material phases (solid, liquid and gas) can be distinguished in the X-ray images as regions with different brightness of the pixels, which is known to be proportional to the local matter density^28^. Examples of the solid, liquid and gas phases are indicated in Fig. 3A, *t* = 5 ms by blue, orange and red arrows, respectively. Additionally, the borders between phases can be seen as dark pixels due to the refraction of the electrons at the boundaries^28^. An example of this phenomenon is shown in Fig. 3A, *t* = 5 ms where the border between liquid and solid phases is indicated with a black arrow.

The following description of the laser process shown in Fig. 3A, is in time order with *t* = 0 ms corresponding to the start of the laser emission. The beginning of the process was characterized by a small portion of the incoming laser energy being absorbed due to the high reflectivity of aluminum in solid phase^40^. The reflectivity decreased with the temperature rise^40^, leading to the formation and expansion of the melt pool during further exposure. The melt pool became observable with a few milliseconds delay (Fig. 3A, *t* = 3 ms). At this stage, the geometry of the melt pool was defined by the heat transfer, which is well predictable and is a characteristic of conduction welding *-* one of the major industrial welding regimes^3^. The advantage of this weld regime is in the high reproducibility of weld quality, mostly due to the stable dynamics of the melt pool^3^.

As the process continued, the melt pool’s temperature kept rising until reaching the boiling point of aluminum. This induced evaporation of the molten liquid, which increased with further energy deposition. When recoil pressure from the evaporation became sufficiently strong, the melt pool was pushed aside, forming a narrow capillary known as a keyhole (Fig. 3A, *t* = 3.5 ms)^3,11,41^. In a stable state (Fig. 3A, *t* = 3.5 ms), the keyhole was characterized by a cone-shaped channel, which was wider near the surface and narrowed in depth^11,20,42–44^. As it contains mostly overheated material vapor, the laser beam can propagate deeper inside the material. At the same time, the laser absorption is significantly enhanced via multiple reflections of the beam between the keyhole’s wall^45^. Consequently, this weld regime allows achieving a deep weld with high aspect ratio, making it popular in industrial welding^3^. Another advantage of the keyhole welding is that it can provide better material fusion between the counterparts due to the higher dynamics of mass flow inside the melt pool as compared to conduction welding. In this case, this mass flow is strongly dependent on multiple factors, such as recoil pressure, surface tension and Marangoni effect^3,6,11,19,41^.

Despite the advantages of keyhole weld regime, it is prone to defects caused by the instability of the keyhole channel, which increases with greater depth. Unstable keyhole can be observed in the experiment shown in Fig. 3A starting from *t* = 5 ms. It is characterized by geometrical fluctuations of the keyhole channel. This phenomenon can be explained by the fact that a stable keyhole requires a balance between several factors, where the main ones are the surface tension and recoil pressure^11^. The former is responsible for maintaining the keyhole channel, while the latter for its collapse. It was shown that high keyhole depth leads to increased surface tension while the recoil pressure is reduced^11,42^. Once the surface tension can no longer be compensated, the keyhole channel starts to fluctuate geometrically, leading to fluctuations and irregularities also in the distribution of the absorbed laser energy. This behavior results in non-uniformities in the evaporation and flow dynamic of the molten material, i.e. increased instability of the process^8,11,20^. Events such as blowout, pore formation and spattering can occur as consequences of this weld regime^3,11,20,42,43^. Blowout, defined as the expulsion of molten material from the melt pool, and pore formation can be seen in Fig. 3A at *t* = 6 ms and *t* = 9 ms, respectively. The expelled material and the pore formed during this regime are indicated by red arrows. Detailed descriptions of those phenomena can be found in other works^3,20,46^.

In literature, the instability of the keyhole is considered as a major source of porosity due to the high probability of vapor getting trapped^11,20,47^. However, in the present work, the pores that formed during an unstable keyhole mostly merged with the keyhole channel and disappeared (Fig. 3A, *t* = 9 ms). This behavior can be explained by the fact that the velocity used in our work (1.5 mm/s) was much lower than the velocity used in real welding applications, which is typically hundreds of millimeters per second^3,47^. In our experimental condition, the pores could not travel far away from the fluctuating keyhole channel and, therefore, were prone to merging with the channel. Another typically reported source of porosity is by the fast collapse of the keyhole channel at the end of the process, which often leads to vapor being trapped in the melt pool and forming pores^11,48,49^. This phenomenon was also observed in our work and can be seen in Fig. 3A, *t* = 10–10.5 ms. The resulting pore is marked by a black arrow.

The irregularities in the keyhole are further demonstrated in the graph shown in Fig. 3B. In this figure, the X-ray image on the left is a snapshot at *t* = 7 ms and the plot on the right displays the variation of pixel brightness with depth along the keyhole channel, as indicated by a red line in the X-ray image. The brightness data were obtained from the X-ray image using ImageJ software^50^. As the pixel intensity is directly related to local matter density^20,24,28^, the plot also indicates irregularities in the evaporation and flow dynamics of the molten material even in stable radiation conditions. Interestingly, the result shown in Fig. 3B implies that the monitoring methods by surface inspection such as with optical sensors and cameras might suffer inaccuracies due to the in-depth irregularities of the keyhole channel.

**Figure 4:**
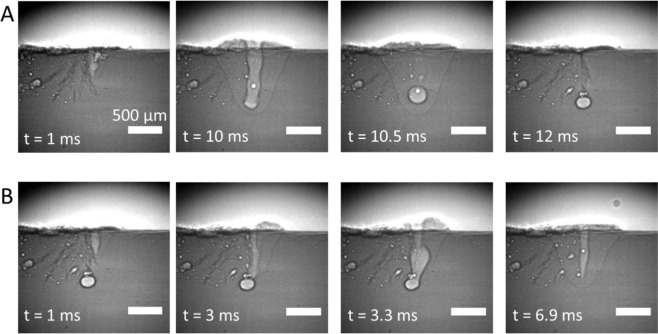
X-ray radiographic images of pore formation (**A**) and removal (**B**) during two successive laser pulses. The formation was caused by the rapid collapse of the keyhole channel, leading to vapor being trapped. On the other hand, the removal of the pore was by merging with the keyhole formed by the following laser pulse. Both laser pulses were of 1 kW power and 10 ms pulse duration. The sample was translated relatively to the laser beam and the X-ray beam at a constant velocity of 1.5 mm/s. The timestamp starts simultaneously with the laser illumination. The marker in the right bottom corresponds to a distance of 500 µm.

Another interesting event, namely the removal of the pores, was also observed during the experiments. Figure 4 shows the X-ray radiographic images of two successive laser pulses in another experiment with 10 ms pulse duration and 1 kW laser power. The formation of a pore at the end of the first laser pulse (Fig. 3A) can be seen at *t* = 10.5–12 ms. Interestingly, the pore merges with the keyhole channel formed by the second pulse and disappear, as shown in Fig. 3B, at *t* = 3.3–6.9 ms. This observation indicates the possibility to repair a porous weld, which has significant importance for the application of laser welding in industrial manufacturing. This phenomenon was also reported by Zhao *et al*.^51^ by *post mortem* inspection of a workpiece subjected to two successive laser weld scans.

The definition of categories for the real-time quality monitoring was carried out with respect to two factors: (i) the laser welding regime and (ii) the presence of defects (i.e. blowout, porosity). Both factors are known to be critical for the mechanical properties of the weld joint and are widely used for characterization in industrial validation tests^3,52^. Consequently, the following categories were considered in the present work: *conduction welding*, *stable keyhole, unstable keyhole*, *blowout* and *pores*. While the definitions of *conduction welding* and *stable keyhole* are straightforward, the definitions of the other three categories require further explanations. *Unstable keyhole* was defined as a stage of the process in which the keyhole channel was fluctuating without the occurrence of events such as *blowout* and *pore formation*. The *unstable keyhole* category is of particular interest in real-time monitoring of the process as it is prone to undesirable events that lead to defects. *Blowout* was considered to start at the beginning of the material expulsion and end when the molten material fell back to the surface. The category *pores*, on the other hand, was assigned to the appearance of pores by highly unstable keyhole (Fig. 3A, *t* = 9 ms). As mentioned in the previous paragraph, most of the pores formed during this stage merged with the keyhole channel almost immediately due to the experimental limitations in the present work. Consequently, the merging of the pores could not be separated and was, therefore, included in this category.

### LBR and AE signatures for laser welding

**Figure 5:**
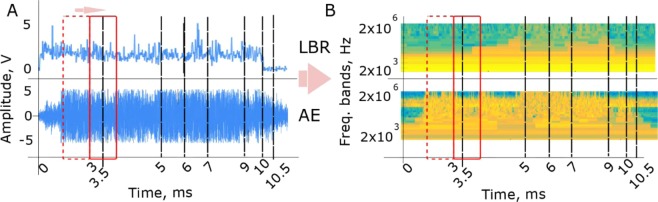
(**A**) LBR (top) and AE (bottom) signals of the laser welding from Fig. 3; (**B**) the wavelet spectrograms for LBR (top) and AE (bottom) signals from (**A**). Both spectrograms were obtained applying wavelet packet transform^38^ with Daubechies wavelet of ten vanishing moments^36^. The relative energies of the frequency bands were computed according to^35,36^. The timestamps in (**A**) correspond to events in Fig. 3, (**A)**.

The corresponding LBR and AE signals for the experiment in Fig. 3A are shown in Fig. 5A. The timestamps in Fig. 5 are synchronized with the radiographic images from Fig. 3A so that both data can be put in correspondence. It can be seen that the LBR and AE signals evolve differently during the process, implying that they are sensitive to different physical phenomena. In particular, the beginning and the end of the process could be clearly recognized in the LBR, as they are characterized by a sharp increase and decrease, respectively, of the signal. That behavior is understandable as the Ge photodiode measured mainly the amount of laser energy being reflected. The other events, especially *blowout*, surprisingly cannot be recognized just by visual inspection of the signal.

On the other hand, the AE signal exhibits only a small increase in its amplitude as the process starts. It can be explained by the relationship between the AE strength and the size of the melt pool. Another difference between the LBR and AE is that the latter does not drop immediately as the radiation stops. Instead, it reduced gradually together with the shrinking of the melt pool. Similarly to the LBR, the quality-significant events cannot be clearly recognized in the AE signal. Similar observations and conclusions were reported in our previous works^29,31^.

Interestingly, the interpretation of the process using wavelet spectrogram provides better precision. This is particularly true from the start of the process up to the formation of the keyhole channel (Fig. 3A and Fig. 5B, top, *t* < 3.5 ms), which is mainly characterized by the presence of low frequencies in the LBR signal. In contrast, the occurrence of the *stable keyhole* (Fig. 5B, top, 3.5 ms ≤ *t* ≤ 5 ms) is characterized by the appearance of higher frequency contents. These same high frequency contents can be also observed during *unstable keyhole* (Fig. 5B, top, 7 ms ≤ *t* ≤ 9 ms) whereas *blowout* (Fig. 5B, top, *t* = 6 ms) can be seen as a temporary attenuation of the specific frequencies.

The spectrogram of the AE signal (Fig. 5B, bottom) also exhibits low frequency content at the beginning of the process. Similar to the LBR signals, the higher frequency content appears as the process becomes more complex and unstable. Interestingly, no attenuation of the high frequency content is observed during *blowout* as in the case of the LBR spectrogram.

While better information can be obtained from the spectrograms, it is challenging to recognize all the events and categories. In addition, real-time classification of the quality-significant events is not feasible via visual inspection. Therefore, the detailed analysis of the structure in the spectrograms was dedicated to CNN. As described in Fig. 2B, the search of stable combinations of frequency bands (the vertical components of the spectrogram) and their evolution in time (the horizontal component of the spectrogram) was the target for pattern self-learning within the CNN. The structure of the database that is sufficient for this task is given in the materials and methods section. The complete dataset was divided into two datasets without common signals: training and test sets. This imitates the classification of the unique signatures in real-life signals by an already trained CNN, when new data are collected and no X-ray information is accessible.

**Figure 6:**
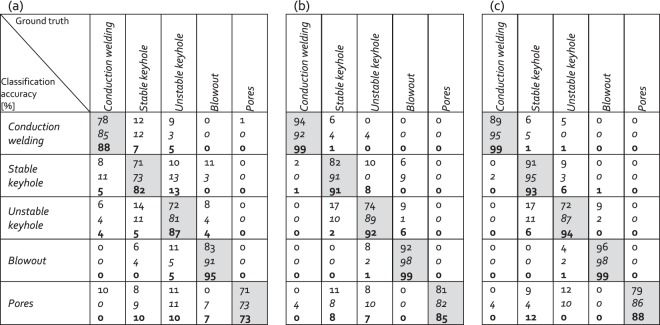
Tables with the results of the accuracy of the classifications for (**a**) the LBR sensor, (**b**) the AE sensor and (**c**) the combination of the two sensors. The classification results in each cell are presented in the following descending order: conventional CNN, *cross residual CNN* (in i*talic)* and **temporal CNN** (in **bold**).

The results of the test classification are presented in Fig. 6. Figure 6(a,b) present the classification using features obtained from the LBR and AE signals, respectively. Meanwhile, Fig. 6(c) displays the results obtained using a combination of features from both signals. The training and test datasets consist of three hundred and one hundred patterns, respectively. The classification accuracies are defined as the number of true positives divided by the total number of tests^22^. These values are given in the diagonal cells of the tables that are filled with grey. Similarly, the classification errors are the ratio of the true negatives divided by the total number of the tests. The corresponding values are in the non-diagonal row cells. Additionally, each table displays the results obtained with three different CNN algorithms. The results are arranged in following descending order: conventional CNN, *cross residual CNN* (*italic*) and **temporal CNN** (**bold**). For example, for the LBR (see Fig. 6(a)), the classification results for the category *unstable keyhole* was classified with an accuracy rate of 87% using temporal CNN (**bold red**). The errors are due to an overlap with the categories *conduction welding, stable keyhole* and *keyhole explosion* with error rates of 4% (**bold blue**), 5% (**bold green**) and 4% (**bold black**), respectively.

The classification results displayed in Fig. 6 are within the range 71–99%. It confirms the existence of unique signatures in the LBR and AE signals of the quality-significant events, which could be extracted using our signal processing approach. Among the tested CNN algorithms, best classification rates were obtained with temporal CNN. It indicates the importance of the choice of a proper processing framework. Within this investigation, the discussions and the general recommendations of CNN tuning were outside the scope, but this work is planned as the future continuation of this study.

A more detailed inspection of Fig. 6 shows that the AE signals generally give better classification accuracy than the LBR. This is consistent with the results of our previous work^22^. It might be explained by the different natures of the signals. The AE senses a volumetric fluctuation of the melt pool and/or vapor channel of the keyhole while the LBR detect/measure variations of the laser reflection at the melt pool’s surface. The latter sensing technique relies on a good correlation between the in-depth fluctuation of the melt pool and the surface distortion, which seems to decrease at increasing depth, leading to worse classification^22^. Another surprising finding is the low accuracy of the *conduction welding* with significant misclassification rates shared between *stable keyhole* and *unstable keyhole* even though the differences in the surface behavior of those categories are obvious (Fig. 3A). It implies that another reason for the low classification accuracy of LBR signal must be also considered. In particular, it is reasonable to expect that the LBR signal suffers from its low acquisition rate (50–2,000 kHz) as compared to the AE acquisition rate (10,000 kHz). In this condition, the high frequency contents of the LBR signal, which might be useful for the classification, might have been filtered. Additional works need to be done to confirm this reasoning.

On the contrary to the LBR signal, the AE seems not affected significantly by the increasing depth of the melt pool. A classification rate of as high as 92% is obtained for *unstable keyhole*, only slightly lower than that of *conduction welding* while *stable keyhole* can be classified at a confidence level as high as 91%. The classifications of *blowout* and *pores* also reach high accuracies, of 99% and 85%, respectively. While the former category is mostly misclassified with *unstable keyhole*, the latter is with both *unstable keyhole* and *stable keyhole*, equally, indicating that there are overlapping in features corresponding to those categories. The very good classification results obtained with the AE signals show that the unique features of the consider events can be separated well with our signal processing approach. A slight improvement of the classification is obtained with a combination of LBR and AE features. The observation implies that the LBR features do contain complementary information about the process. In addition, the use of several measurement sources at the same time should be able to provide good stability for the unique signal features, which is needed in real-life noisy environment. Therefore, we believe that the unification of optical and AE sensors would be advantageous for industrial process monitoring. This issue will be addressed in future work.

An important result is shown in Fig. 6. It is the classification of *stable keyhole* and *unstable keyhole* with high level of confidence (93% and 94%, respectively, with the unification of LBR and AE). As the latter category is known to be prone to defects, its detection in real-time is highly desirable. However, this task has not been accomplished in any other previous research works^13,21^, probably due to the smooth transition between the *stable keyhole* and *unstable keyhole*, making their signature features close to each other^22^. The transition can be provoked easily even in constant irradiation conditions by variations of the local physical properties of the workpiece^42^. The good classification results obtained in the present work are the basis of a fully automatic closed control loop for a defect-free process, the next target of our research.

**Figure 7:**
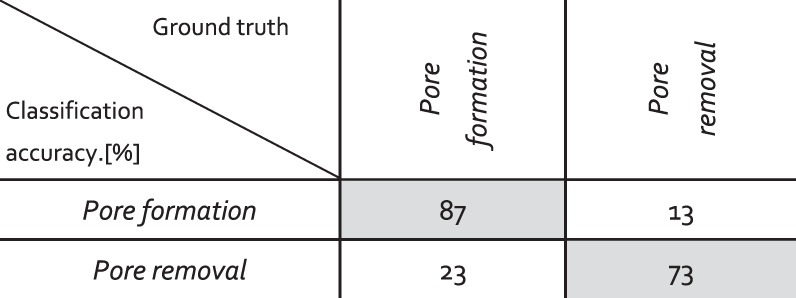
Table with the results of the classifications accuracy for *pore formation* and *pore removal* using temporal CNN.

Another issue of high interest for the real-time monitoring and control of the welding process is the capability to detect the formation and removal of pores. While the former allows determining the location of the defect, which is not always avoidable, the latter allows efficiently removing the defect to repair the defective weld^51^. Unfortunately, as mentioned in the previous section, it was not possible in the present work to separate the formation and removal of pores during an unstable keyhole, due to the very short lifetime of the pores. Therefore, to test the performance of our approach on this issue, two additional categories are taken into consideration. The first one is *pore formation*, assigned to the rapid collapse of the keyhole at the end of the process, leading to trapping of vapor and hence, formation of pores (Fig. 4A, *t* = 10–10.5 ms). It must be emphasized that not all keyhole collapse leads to pore formation, therefore, only those with remaining pores are considered. In addition, as this category is assigned to the end of the process, there is no LBR signal available. The second category is *pore removal*, which includes the interaction between the keyhole and the existing pores, leading to the removal of the latter (Fig. 4B). The classification of those two categories was done using temporal CNN, which gave the best results for the previous classifications (Fig. 6). Each category consists of thirty samples for training and ten for testing. The results are shown in Fig. 7. They were calculated in a similar manner as those shown in Fig. 6. The p*ore formation* and *pore removal* categories can be classified with decent confidence levels of 87% and 73%, respectively. The results are rather impressive considering the sizes of our datasets, which are very small compared to machine learning standards. It indicates the robustness of our approach. In addition, it implies that there are distinct differences in the extracted features for those two categories. The link between the signal features and the underlying physical phenomena will be explored in future work.

## Conclusion

The present work demonstrates an innovative approach for monitoring in real-time the events that have a significant influence on the laser welding quality. The approach involves the use of various sensors and a state-of-the-art machine learning approach for signal processing. Additionally, the dynamics of the process was visualized by X-ray radiographic imaging of the process zone. From the X-ray data, the following events are considered *conduction welding*, *stable keyhole, unstable keyhole*, *blowout* and *pores*. They were classified with accuracies within the range 71–99%, indicating the good performance of our signal processing approach.

There are three important results from the present work. Firstly, our approach is capable of distinguishing between *stable keyhole* and *unstable keyhole* with very high confidence. Since the latter regime is prone to defects, the capability to detect the stable – unstable keyhole transition is of high interest. It is a prerequisite for the realization of a closed-loop control for the process to avoid defects. Secondly, it is possible to distinguish between the formation and removal of pores, one of the most dangerous defects. This result is interesting as it allows monitoring the removal of defects in welds, which are not always avoidable. Thirdly, it was shown that the unification of LBR and AE features could improve the classification accuracy. Though the improvements observed in the present work are not significant, it is probably due to the fact that the LBR signals were acquired at a rather low acquisition rate.

It must be emphasized that such a good classification accuracy was achieved using only LBR and AE sensors, which are cheap and easy to implement and integrate into an existing industrial environment. The classification results can even be improved further by increasing the size of the dataset. Additionally, optimizing the machine learning algorithm is also a useful method.

Besides the good classification rates, other strong points of the present approach are the real-time performance and the readiness for industrial implementation. Firstly, the computation for each classification requires as low as 2 ms, indicating a possibility to follow the process in real-time. Secondly, the low cost embedding’s of algorithms into the parallel computing hardware makes such systems ready for real-life usage. In the practical application of this approach, the X-radiographic observation only needs to be used once to obtain a library of features for different regimes/events of interest. Afterward, the real-time monitoring system can operate with simple and low cost sensing techniques as proposed in the present work. In order to study the generalization capabilities of the monitoring system, the possibility to apply the model trained on one material to others will be addressed in future works. It must be emphasized that this approach is also a potential solution for monitoring other laser-based processes such as additive manufacturing, laser drilling and laser shock peening.

The scientific value of our approach is in the recovery of complex responses of the LBR and AE signals to the underlying physical phenomena using machine learning. The discovered signatures in LBR and AE may help in a better understanding of the behavior of the liquid/gas phases of the material. The interpretations of those signatures in terms of the underlying physics (pressures fluctuations for AE and surface distortions in LBR) are planned as a continuation of this work.

## Materials and Methods

### Welding samples material

2 mm thick plates of AA5005 aluminum alloy (98.1%Al, 0.9% Mg and 1% of other elements) were used. Thermal conductivity of the material is 237 W/(m.K) at 293 K and the melting point is approximately 933.3 K.

### Sample holder

The same material as samples.

### Laser source and laser delivery system

A fiber laser system Starfiber 150/300P (Coherent Inc., Switzerland) was used. The emission wavelength and the maximum peak laser power were 1070 nm and 1.5 kW, respectively. The delivery of the light from the source to the laser head was carried out through a single mode fibre of 12 µm core diameter. The laser head was a customized version of the FLBK 60 model from Coherent Inc., Switzerland. The focal length of the output focusing lens was 170 cm. The laser head was additionally equipped with a beam splitter for the detection of laser back reflection.

### Acoustic emission and optical sensors

The detection of the laser back reflection (LBR) was provided by a Ge photodiode with the original sensitivity in a spectral range (800–1800) nm. It was equipped with a narrow band pass optical filter at the laser wavelength for selective transmission of the reflected laser radiation. The LBR signal acquisition was by an oscilloscope HDO 6104 (Lecroy, USA). The sampling rate varied in the range (50–2000) kHz, depending on the duration of the whole experiment. The acoustic emission (AE) was detected by piezo sensor PICO HF-1.2 (Physical Acoustics, Germany) and recorded with Vallen system (Vallen, Germany) with a sampling rate of 10 MHz.

### Synchrotron hard X-ray imaging

The scintillator detector converted the incoming beam with the wavelength of (0.5 Å) to the visible spectral range of 550 nm. The re-emitted luminescence was directed to a high-speed camera pco.Dimax (PCO AG, Germany), operating at a framerate 28,762 fps and an exposition time of 30 µs.

### Welding experiments

The laser welding experiments were carried out in pulsed mode with a repetition rate of 10 Hz, pulse durations and output laser powers within the ranges 10–15 ms and 1–1.5 kW, respectively. The combinations of these process parameters were chosen to provoke different welding qualities. Thirty five welding experiments were performed.

### Computational station

The configuration of the computational station was based in two Intel Xeon E5-2630 v4 2.2 GHz Processors with 10 cores, 64GB DDR4 RAM and operation frequency 2133 MHz. The station was additionally equipped with two nVidia Tesla P100 graphic cards with 12GB memory on each and double precision of 64 bit. The parallel computing library NVidia Cuda 8.0 was used and the computations were operated by 64 bit CentOS 7.4. The coding was performed in Visual Studio 2017 using C# and Python 3.6.

### Deep learning libraries and custom software development tools

PyTorch 0.4.6^53^.

### Deep learning network

Conventional CNN structure: conv(relu, 3 × 4), pool(2), conv(relu), pool(2), conv(relu), pool(2), conv(relu), pool(2), conv(relu), pool(2), conv(relu), pool(2), fullc(), fullc(), softmax.

### Training details

100 epochs, learning rate: 0.001, optimizer: Adam, no regularizers applied.

### Datasets

The training and test datasets included three hundred and one hundred, respectively, patterns for each category.
