# Author Correction: Supervised deep learning for real-time quality monitoring of laser welding with X-ray radiographic guidance

**DOI:** 10.1038/s41598-020-65966-2

**Published:** 2020-06-04

**Authors:** Sergey Shevchik, Tri Le-Quang, Bastian Meylan, Farzad Vakili Farahani, Margie P. Olbinado, Alexander Rack, Giulio Masinelli, Christian Leinenbach, Kilian Wasmer

**Affiliations:** 1 Laboratory for Advanced Materials Processing (LAMP), Swiss Federal Laboratories for Materials Science and Technology (Empa), Thun, Switzerland; 2 Coherent Switzerland, Belp, CH-3125 Switzerland; 3 ESRF – The European Synchrotron, Grenoble, France

Correction to: *Scientific Reports* 10.1038/s41598-020-60294-x, published online 25 February 2020

This Article contains typographical errors in the Results and Discussion section.

Under subheading ‘Definition of the quality-significant events using X-ray radiography’,

“It is characterized by geometrical fluctuations of the keyhole channel. This phenomenon can be explained by the fact that a stable keyhole requires a balance between several factors, where the main ones are the surface tension and recoil pressure^11^. The former is responsible for maintaining the keyhole channel, while the latter for its collapse. It was shown that high keyhole depth leads to increased surface tension while the recoil pressure is reduced^11,42^.”

should read:

“It is characterized by geometrical fluctuations of the keyhole channel. This phenomenon can be explained by the fact that a stable keyhole requires a balance between several factors, where the main ones are the recoil pressure and surface tension^11^. The former is responsible for maintaining the keyhole channel, while the latter for its collapse. It was shown that high keyhole depth leads to increased surface tension while the recoil pressure is reduced^11,42^.”

“The formation of a pore at the end of the first laser pulse (Fig. 3A) can be seen at *t*  =  10.5–12 ms. Interestingly, the pore merges with the keyhole channel formed by the second pulse and disappear, as shown in Fig. 3B, at *t*  =  3.3–6.9 ms.”

should read:

“The formation of a pore at the end of the first laser pulse (Fig. 4A) can be seen at *t*  =  10.5–12 ms. Interestingly, the pore merges with the keyhole channel formed by the second pulse and disappear, as shown in Fig. 4B, at *t*  =  3.3–6.9 ms.”

Additionally, under subheading ‘LBR and AE signatures for laser welding’,

“For example, for the LBR (see Fig. 6(a)), the classification results for the category *unstable keyhole* was classified with an accuracy rate of 87% using temporal CNN (**bold red**). The errors are due to an overlap with the categories *conduction welding, stable keyhole and keyhole explosion* with error rates of 4% (**bold blue**), 5% (**bold green**) and 4% (**bold black**), respectively.”

should read:

“For example, for the LBR (see Fig. 6(a)), the classification results for the category *unstable keyhole* was classified with an accuracy rate of 87% using temporal CNN. The errors are due to an overlap with the categories *conduction welding, stable keyhole and keyhole explosion* with error rates of 4%, 5% and 4%, respectively.”

